# Supplementary material for: Job satisfaction as a moderator between organizational culture and turnover intention among Malaysian police officers
Source: PLoS One. 2025 Sep 2;20(9):e0331458. doi: 10.1371/journal.pone.0331458 (PMC12404360; doi:10.1371/journal.pone.0331458)
Supplement: Attachment — Submitted filename: PONE-D-25-23324_reviewer Budiman.pdf [file pone.0331458.s001.pdf]

PLOS ONE

EXPLORING THE INFLUENCE OF ORGANIZATIONAL CULTURE AND JOB SATISFACTION ON TURNOVER INTENTIONS AMONG POLICE OFFICERS : A MODERATION ANALYSIS

--Manuscript Draft--

**Manuscript Number:**	PONE-D-25-23324
**Article Type:**	Research Article
**Full Title:**	EXPLORING THE INFLUENCE OF ORGANIZATIONAL CULTURE AND JOB SATISFACTION ON TURNOVER INTENTIONS AMONG POLICE OFFICERS : A MODERATION ANALYSIS
**Short Title:**	EXPLORING THE INFLUENCE OF ORGANIZATIONAL CULTURE AND JOB SATISFACTION ON TURNOVER INTENTIONS
**Corresponding Author:**	Erny Yusnida Yusnida binti Che Che Yusoff<br>National University of Malaysia: Universiti Kebangsaan Malaysia<br>KAJANG, None Selected MALAYSIA
**Keywords:**	organizational culture, job satisfaction, turnover intention, police officers, moderation analysis, employee retention
**Abstract:**	This study investigates the relationships among organizational culture, job satisfaction, and turnover intentions among police officers in Malaysia, with a specific focus on whether job satisfaction moderates the effect of organizational culture on turnover intention. Given the critical role of organizational culture and job satisfaction in shaping employee retention, this research aims to provide insights into how these factors interact to influence turnover intentions in a high-stress, high-demand profession like law enforcement. Data were collected from 373 police officers across Malaysia and analyzed using a moderated regression model with SPSS Process Macro. The findings indicate that both organizational culture and job satisfaction independently predict turnover intention, with stronger organizational culture and higher job satisfaction associated with lower turnover intentions. However, the moderation analysis revealed that job satisfaction does not significantly moderate the relationship between organizational culture and turnover intention, suggesting that the effect of organizational culture on turnover intentions remains consistent regardless of job satisfaction levels. These results underscore the importance of fostering a supportive organizational culture to reduce turnover intentions, while also suggesting that job satisfaction alone may not alter the impact of organizational culture on turnover. This study contributes to the understanding of turnover dynamics in law enforcement and offers practical implications for police management in developing effective retention strategies.
**Order of Authors:**	Erny Yusnida Yusnida binti Che Che Yusoff
	Mohd Nasir Selamat
	Rusyda Helma Mohd
**Additional Information:**	
**Question**	**Response**
**Financial Disclosure**<br><br>Enter a financial disclosure statement that describes the sources of funding for the work included in this submission. Review the submission guidelines for detailed requirements. View published research articles from *PLOS ONE* for specific examples.	The author(s) received no specific funding for this work.

Powered by Editorial Manager® and ProduXion Manager® from Aries Systems Corporation

This statement is required for submission and **will appear in the published article** if the submission is accepted. Please make sure it is accurate.<br><br>**Funded studies**<br>Enter a statement with the following details:<br>• Initials of the authors who received each award<br>• Grant numbers awarded to each author<br>• The full name of each funder<br>• URL of each funder website<br>• Did the sponsors or funders play any role in the study design, data collection and analysis, decision to publish, or preparation of the manuscript?<br><br>Did you receive funding for this work?	
**Competing Interests**<br><br>Use the instructions below to enter a competing interest statement for this submission. On behalf of all authors, disclose any competing interests that could be perceived to bias this work—acknowledging all financial support and any other relevant financial or non-financial competing interests.<br><br>This statement is required for submission and **will appear in the published article** if the submission is accepted. Please make sure it is accurate and that any funding sources listed in your Funding Information later in the submission form are also declared in your Financial Disclosure statement.<br><br>View published research articles from *PLOS ONE* for specific examples.	NO authors have competing interests

**NO authors have competing interests**<br><br>Enter: *The authors have declared that no competing interests exist.*<br><br>**Authors with competing interests**<br><br>Enter competing interest details beginning with this statement:<br><br>*I have read the journal's policy and the authors of this manuscript have the following competing interests: [insert competing interests here]*<br><br>* typeset	
**Ethics Statement**<br><br>Enter an ethics statement for this submission. This statement is required if the study involved:<br><br>• Human participants<br>• Human specimens or tissue<br>• Vertebrate animals or cephalopods<br>• Vertebrate embryos or tissues<br>• Field research<br><br>Write "N/A" if the submission does not require an ethics statement.<br><br>General guidance is provided below. Consult the submission guidelines for detailed instructions. **Make sure that all information entered here is included in the Methods section of the manuscript.**	Human Subject Research (involving human participants and/or tissue)

Powered by Editorial Manager® and ProduXion Manager® from Aries Systems Corporation

**Format for specific study types**<br><br>**Human Subject Research (involving human participants and/or tissue)**<br>• Give the name of the institutional review board or ethics committee that approved the study<br>• Include the approval number and/or a statement indicating approval of this research<br>• Indicate the form of consent obtained (written/oral) or the reason that consent was not obtained (e.g. the data were analyzed anonymously)<br><br>**Animal Research (involving vertebrate animals, embryos or tissues)**<br>• Provide the name of the Institutional Animal Care and Use Committee (IACUC) or other relevant ethics board that reviewed the study protocol, and indicate whether they approved this research or granted a formal waiver of ethical approval<br>• Include an approval number if one was obtained<br>• If the study involved *non-human primates*, add *additional details* about animal welfare and steps taken to ameliorate suffering<br>• If anesthesia, euthanasia, or any kind of animal sacrifice is part of the study, include briefly which substances and/or methods were applied<br><br>**Field Research**<br><br>Include the following details if this study involves the collection of plant, animal, or other materials from a natural setting:<br>• Field permit number<br>• Name of the institution or relevant body that granted permission	
**Data Availability**<br><br>Authors are required to make all data underlying the findings described fully available, without restriction, and from the time of publication. PLOS allows rare exceptions to address legal and ethical concerns. See the PLOS Data Policy and FAQ for detailed information.	Yes - all data are fully available without restriction

Powered by Editorial Manager® and ProduXion Manager® from Aries Systems Corporation

A Data Availability Statement describing where the data can be found is required at submission. Your answers to this question constitute the Data Availability Statement and **will be published in the article**, if accepted.<br><br>**Important:** Stating 'data available on request from the author' is not sufficient. If your data are only available upon request, select 'No' for the first question and explain your exceptional situation in the text box.<br><br>Do the authors confirm that all data underlying the findings described in their manuscript are fully available without restriction?	
**Describe where the data may be found in full sentences. If you are copying our sample text, replace any instances of XXX with the appropriate details.**<br><br>• If the data are **held or will be held in a public repository**, include URLs, accession numbers or DOIs. If this information will only be available after acceptance, indicate this by ticking the box below. For example: *All XXX files are available from the XXX database (accession number(s) XXX, XXX.*).<br>• If the data are all contained **within the manuscript and/or Supporting Information files**, enter the following: *All relevant data are within the manuscript and its Supporting Information files.*<br>• If neither of these applies but you are able to provide **details of access elsewhere**, with or without limitations, please do so. For example:<br><br>*Data cannot be shared publicly because of [XXX]. Data are available from the XXX Institutional Data Access / Ethics Committee (contact via XXX) for researchers who meet the criteria for access to confidential data.*<br><br>*The data underlying the results presented in the study are available from (include the name of the third party*	The Data are our own collection.

Powered by Editorial Manager® and ProduXion Manager® from Aries Systems Corporation

and contact information or URL).<br>• This text is appropriate if the data are owned by a third party and authors do not have permission to share the data.<br><br>* typeset	

EXPLORING THE INFLUENCE OF ORGANIZATIONAL CULTURE AND JOB SATISFACTION ON TURNOVER INTENTIONS AMONG POLICE OFFICERS : A MODERATION ANALYSIS

Commented [BB1]: The title is informative but could be more concise. It also does not clearly indicate the moderating role being tested.
suggestion:
Consider revising it to something like. *The Role of Job Satisfaction in the Relationship Between Organizational Culture and Turnover Intention Among Malaysian Police Officers: A Moderated Regression Analysis.*

Erny Yusnida binti Che Yusoff [1], Dr Mohd Nasir bin Selamat [2], Dr Rusyda Helma binti Mohd [3]

[1] Faculty of Social Science and Humanities, The National University of Malaysia

[2] Faculty of Social Science and Humanities, The National University of Malaysia

[3] Faculty of Social Science and Humanities, The National University of Malaysia

ABSTRACT

This study investigates the relationships among organizational culture, job satisfaction, and turnover intentions among police officers in Malaysia, with a specific focus on whether job satisfaction moderates the effect of organizational culture on turnover intention. Given the critical role of organizational culture and job satisfaction in shaping employee retention, this research aims to provide insights into how these factors interact to influence turnover intentions in a high-stress, high-demand profession like law enforcement. Data were collected from 373 police officers across Malaysia and analyzed using a moderated regression model with SPSS Process Macro. The findings indicate that both organizational culture and job satisfaction independently predict turnover intention, with stronger organizational culture and higher job satisfaction associated with lower turnover intentions. However, the moderation analysis revealed that job satisfaction does not significantly moderate the relationship between organizational culture and turnover intention, suggesting that the effect of organizational culture on turnover intentions remains consistent regardless of job satisfaction levels. These results underscore the importance of fostering a supportive organizational culture to reduce

Commented [BB2]: Comment:
The abstract lacks a clear structure. It should follow the standard format: background, objective, methods, results, and conclusion.
Suggestions:
Clearly state the research problem.
Mention the sample size and method of analysis.
Clarify whether the moderation hypothesis was supported or not.
Avoid overly technical language to ensure accessibility.
Example Addition: Using moderated regression analysis via SPSS Process Macro, this study found that both organizational culture and job satisfaction were significantly related to turnover intention. However, job satisfaction did not moderate the relationship between organizational culture and turnover intention.

turnover intentions, while also suggesting that job satisfaction alone may not alter the impact of organizational culture on turnover. This study contributes to the understanding of turnover dynamics in law enforcement and offers practical implications for police management in developing effective retention strategies.

Keywords: organizational culture, job satisfaction, turnover intention, police officers, moderation analysis, employee retention

1. INTRODUCTION

In the law enforcement sector, job satisfaction and organizational culture are critical factors influencing officers' intention to remain within their organizations. Recent studies underscore that organizational culture plays a significant role in shaping employees' commitment, especially in high-stress environments such as policing, where factors like leadership, ethics, and career development can impact morale and retention (Omar et al., 2020; Sulaiman, 2024). For police officers in Malaysia, organizational culture and job satisfaction are closely linked to decisions to leave or stay, as these elements affect work-life balance, stress management, and overall well-being (Francis et al., 2021). Research highlights that when officers perceive their work environment positively and experience job satisfaction, they are more likely to remain committed, thereby reducing turnover intentions (Ismail et al., 2023). Additionally, job satisfaction can act as a moderator, strengthening the relationship between organizational culture and retention by buffering against negative aspects of the job (Narandaran, 2018). Given the significant stressors Malaysian police officers face, examining how job satisfaction mediates between organizational culture and turnover intention is essential for implementing effective retention strategies (Saad & Rashid, 2023). This study, therefore, explores these dynamics within Malaysian policing to provide insights into fostering a supportive and retention-oriented organizational culture.

Commented [BB3]: Comment. The introduction provides some background, but it lacks a coherent flow and fails to clearly identify the research gap and the novelty of the study. Suggestions.

1.Start with a strong opening sentence about the importance of understanding turnover intention in policing.
2.Clearly articulate what previous studies have found and where gaps still exist.
3.Define key terms (e.g., organizational culture, turnover intention) early on.
4.Explicitly state the purpose and objectives of the study.

Example Addition. While prior studies have established links between organizational culture, job satisfaction, and turnover intention, few have explored these relationships within law enforcement contexts in Malaysia, and even fewer have tested for a moderating effect of job satisfaction

Police officers face a demanding work environment characterized by high stress, extensive responsibilities, and frequent exposure to adverse situations. Within such a context, organizational culture and job satisfaction have emerged as critical factors influencing turnover intentions (Sulaiman, 2024). Organizational culture encompasses shared values, beliefs, and practices that shape how individuals behave and interact within an organization. For police officers in Malaysia, a supportive organizational culture - marked by trust, inclusivity, and ethical leadership - can significantly impact their job satisfaction, thereby reducing turnover intentions (Ghazali & Asmawi, 2020; Jabeen et al., 2019).

Job satisfaction, on the other hand, reflects employees' feelings about their roles, work conditions, and relationships within the organization. Satisfied police officers are more likely to demonstrate organizational commitment and lower intentions to leave, even amid the challenges of law enforcement. Studies have indicated that Malaysian police officers who report high job satisfaction are generally more committed to their roles and less inclined to consider other employment opportunities (Ismail et al., 2023). These elements highlight the need for Malaysian police departments to foster a positive organizational culture that enhances job satisfaction and mitigates turnover rates, particularly given the high costs and societal impacts associated with police turnover (Narandaran, 2018).

High turnover rates within the police force are an issue of concern for Malaysian law enforcement agencies, as frequent attrition disrupts organizational stability and incurs significant recruitment and training costs (Hassan et al., 2018). Research indicates that the Royal Malaysian Police (RMP) faces notable turnover rates, largely attributed to issues within organizational culture and job dissatisfaction (Omar et al., 2020). Officers often experience stress and dissatisfaction due to perceived inequities, limited career advancement opportunities, and a lack of supportive leadership. Consequently, many police officers express intentions to

leave, seeking work environments that offer better support, career growth, and alignment with personal values (Saad & Rashid, 2023).

A pressing question arises: how can job satisfaction moderate the influence of organizational culture on police officers' turnover intentions? The moderating role of job satisfaction in this context remains underexplored, despite its potential to buffer the adverse effects of a less supportive culture (Francis et al., 2021). Addressing this gap in understanding is crucial for Malaysian police departments aiming to implement retention strategies that reinforce a positive culture and enhance job satisfaction. Identifying effective interventions could help mitigate turnover intentions among police officers, leading to improved workforce stability and organizational performance.

This study holds significant implications for law enforcement and human resource management within the Malaysian police force. First, by exploring the relationship between organizational culture, job satisfaction, and turnover intentions, it provides valuable insights for designing retention strategies. Understanding how a supportive organizational culture and high job satisfaction reduce turnover intentions can guide policymakers and police leaders in creating a work environment that fosters commitment and resilience among officers (Al-Shurafat & Halim, 2018). Moreover, this study highlights the role of job satisfaction as a moderating factor, suggesting that even in challenging cultural settings, enhancing officers' satisfaction with their roles may help mitigate turnover intentions (Septriyan, 2022).

Commented [BB4]: Comment.
There is no separate section for literature review or theoretical framework. This makes it difficult to assess the conceptual basis of the hypotheses.
Suggestions
1.Create a dedicated section titled "Literature Review" or "Theoretical Framework."
2.Present definitions, theories, and previous findings in a logical sequence. Organizational Culture → Job Satisfaction → Turnover Intention → Moderation Hypothesis
3.Reference relevant models (e.g., Job Demands-Resources Model, Social Exchange Theory).

Further, this research underscores the practical need for Malaysian police departments to focus on ethical leadership, transparency, and equity within organizational culture. By establishing a fair and supportive work culture, law enforcement agencies can enhance officers' satisfaction and retention, thereby reducing turnover-related costs and improving operational efficiency (Kamaluddin et al., 2024). Lastly, the findings from this study could contribute to broader

discussions on employee well-being and retention in high-stress sectors, providing a framework that other professions can adapt to address similar workforce challenges.

Job satisfaction is an essential factor impacting turnover intentions, especially in high-stress professions such as policing. Studies indicate that factors contributing to job satisfaction among police officers include effective leadership, organizational support, work-life balance, and fair performance appraisals (Omar et al., 2020). This notion is consistent with Francis et al. (2021), who emphasize that adequate support structures can mitigate stress and improve job satisfaction in law enforcement roles. Furthermore, Al-Shurafat and Halim (2018) highlighted that the relationship between job satisfaction and turnover intentions is particularly strong in professions where employees encounter daily stressors, such as the police force.

Organizational culture is a significant predictor of job satisfaction and, consequently, turnover intentions. Cultures that foster inclusivity, respect, and clear ethical guidelines are associated with higher job satisfaction and lower turnover intentions among police officers (Sulaiman, 2024; Ghazali & Asmawi, 2020). Jabeen et al. (2019) emphasize that cultures promoting teamwork and ethical behavior can enhance commitment, particularly within Malaysian police forces. In a supportive culture, police officers are more likely to experience higher job satisfaction, reducing their intention to leave the organization (Ismail et al., 2023).

Recent research highlights job satisfaction as a moderating factor in the relationship between organizational culture and turnover intentions. According to Narandaran (2018), job satisfaction can buffer the effects of a less supportive organizational culture, thereby reducing turnover intentions. For instance, when police officers perceive their organization as supportive and fair, their satisfaction with their role enhances, which diminishes their likelihood of seeking employment elsewhere (Saad & Rashid, 2023).

The high turnover rates in Malaysian police forces can be attributed to several organizational and individual factors. Research by Hassan et al. (2018) identified that dissatisfaction with

leadership styles and lack of clear progression paths often lead to turnover among police officers. Similarly, Abo-Murad et al. (2019) discuss that turnover intentions are frequently higher in organizations where there is a perception of unfair treatment and limited growth opportunities. Studies specifically focusing on Malaysian police officers suggest that addressing job satisfaction and fostering a positive organizational culture could significantly reduce turnover (Chong & Mui, 2021).

The work environment also plays a crucial role in job satisfaction and influences turnover intentions. Ebeh and Ohazurike (2022) argue that a positive work environment, characterized by trust and mutual respect, is essential for police officers. Organizational factors, such as a collaborative culture and recognition of individual contributions, have been associated with higher job satisfaction and lower turnover rates (Septriyan, 2022).

Leadership and ethics are pivotal in determining job satisfaction among police officers. Leaders who demonstrate integrity, fairness, and support for their subordinates contribute positively to job satisfaction, which in turn reduces turnover intentions (Hassan et al., 2018). Kamaluddin et al. (2024) emphasize that when police officers perceive their leaders as ethical and just, they are more likely to feel satisfied and committed to the organization.

The reviewed studies provide a comprehensive understanding of the factors influencing job satisfaction, organizational culture, and turnover intentions among Malaysian police officers. Job satisfaction serves as a critical moderator between organizational culture and turnover intentions, suggesting that policy changes targeting improvement in job satisfaction and fostering a positive organizational culture can substantially enhance retention among police officers.

METHODOLOGY

This study employs a quantitative methodology, specifically utilizing a survey-based approach to explore the correlation between job satisfaction, organizational culture, and turnover

Commented [BB5]: Comment.
The methodology section lacks sufficient detail, especially regarding sampling, instrument validation, and statistical assumptions.
Suggestions:
1.Clarify the sampling technique (e.g., stratified random sampling, convenience sampling).
2.Report the response rate (number of questionnaires distributed vs. returned).
3.Describe how instruments were validated (e.g., pilot test, Cronbach's alpha values for each scale).
4.Discuss steps taken to check assumptions (normality, multicollinearity, homoscedasticity).
5.Justify the use of hierarchical regression and PROCESS Macro for moderation analysis

intentions among police officers in Malaysia. A structured questionnaire was distributed to a sample of 373 police officers across various Malaysian states, ensuring a diverse representation of respondents from different regions and departments (Hassan et al., 2018). The survey instrument included validated scales adapted from previous studies on job satisfaction, organizational culture, and turnover intentions. Each variable was measured on a seven-point Likert scale, ranging from “strongly disagree” to “strongly agree,” allowing for standardized responses and facilitating the analysis of correlations between variables (Omar et al., 2020).

To ensure reliability and validity, the instrument underwent a pre-testing phase with a smaller sample of police officers, followed by adjustments based on their feedback. Cronbach's alpha was calculated for each scale, with values exceeding the commonly accepted threshold of 0.70, indicating acceptable internal consistency (Saad & Rashid, 2023). The study’s correlation analysis employed Pearson’s correlation coefficient to evaluate the strength and direction of the relationships between job satisfaction, organizational culture, and turnover intention, supporting hypothesis testing (Narandaran, 2018).

Data analysis was conducted using SPSS software, facilitating both descriptive and inferential statistical analyses to derive meaningful insights. This methodology enables the study to quantitatively assess the hypothesized relationships and provides a robust framework for understanding the factors influencing Malaysian police officers’ job satisfaction and retention intentions.

The objective of this study is to examine the role of job satisfaction as a potential moderator in the relationship between organizational culture and turnover intention among police officers in Malaysia. Specifically, the study aims to determine whether job satisfaction can mitigate the impact of organizational culture on officers' intentions to leave the organization.

Table 1: Effect of Organizational Culture on Turnover Intention with Job Satisfaction as a Moderator

Predictor	B	SE	t	p	LLCI	ULCI
Constant	-2.8346	2.2722	-1.2476	0.2130	-7.3026	1.6334
Organizational Culture	1.1495	0.4546	2.5286	0.0119	0.2556	2.0435
Job Satisfaction	1.0021	0.4050	2.4744	0.0138	0.2057	1.7985
Organizational Culture X Job Satisfaction	-1.1231	0.7083	-1.5712	0.1170	-2.7711	0.0310

Constant: Turnover Intention

The model shows that both organizational culture and job satisfaction have a significant positive effect on turnover intention independently, with p values of 0.0119 and 0.0138, respectively. However, the interaction term is not statistically significant (p = 0.1170), indicating that job satisfaction does not significantly moderate the relationship between organizational culture and turnover intention.

The main effect of organizational culture on turnover intention was significant, b=1.1495, SE = 0.4546, t=2.5286, p=0.0119, 95% CI 0.2556, 2.0435. This suggests that higher levels of organizational culture are associated with increased turnover intention. Similarly, the main effect of job satisfaction was also significant, b=1.0021, SE= 0.4050, t=2.4744 t = 2.4744, p = 0.0138, 95% CI 0.2057, 1.7985. This indicates that higher levels of job satisfaction are related to increased turnover intention, which may reflect specific dynamics within this sample of police officers.

The interaction between organizational culture and job satisfaction did not reach statistical significance, b=−1.1231, SE=0.0783, t=−1.5712, p=0.1170, 95% CI −0.2771, 0.0310. This result suggests that job satisfaction does not significantly moderate the relationship between organizational culture and turnover intention.

The model summary indicates a moderate correlation ($R = 0.4814$) and shows that approximately 23.18% of the variance in turnover intention ($R^2 = 0.2318$) can be explained by the predictors in the model, with a mean square error (MSE) of 1.4628. The model as a whole is statistically significant ($F(3, 369) = 37.1098$, $p < .001$), suggesting that the predictors significantly contribute to explaining turnover intention. However, the interaction effect between organizational culture and job satisfaction on turnover intention is not statistically significant, as indicated by a negligible change in R^2 ($\Delta R^2 = 0.0051$) and a non-significant $F(1, 369) = 2.4687$, $p = 0.1170$. This implies that job satisfaction does not significantly moderate the relationship between organizational culture and turnover intention in this model.

Table 2: Effect of Organizational Culture on Turnover Intention with Job Satisfaction as a Moderator

Interaction Term	R² Change	F	df1	df2	p
Organizational Culture X Job Satisfaction	0.0051	2.4687	1	369	0.1170

Dependent: Turnover Intention

The findings indicate that both organizational culture and job satisfaction are significant predictors of turnover intention independently. However, job satisfaction does not significantly moderate the relationship between organizational culture and turnover intention in this sample of Malaysian police officers. Further research could explore additional factors that might influence this relationship to provide more comprehensive insights into turnover intentions within police forces.

Commented [BB6]: Comment. The results are presented, but the interpretation of the interaction graph (Fig. 1) is unclear and appears contradictory.
Suggestions
1.Clarify why there is a positive association between organizational culture and turnover intention.
2.Ensure the interpretation aligns with the direction of the regression coefficients.
3.Provide a detailed explanation of the simple slopes analysis.
4.Consider adding a table summarizing the regression models.

Interpretation of the Moderation Analysis

Fig 1: Interaction of Moderation

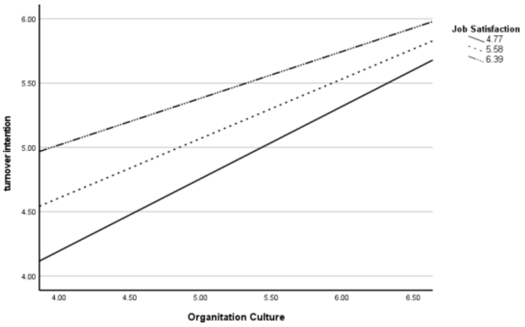

The Fig 1, illustrates the conditional effect of organizational culture on turnover intention at different levels of job satisfaction. Each line represents a different level of job satisfaction (4.77, 5.58, and 6.39 on the seven-point scale).

For all levels of job satisfaction, as organizational culture improves, there is a corresponding increase in turnover intention. This upward trend suggests a positive association between organizational culture and turnover intention within this sample of police officers, potentially indicating that certain aspects of organizational culture may contribute to a desire to leave, or that increased alignment with organizational culture affects perception of turnover differently. While job satisfaction does not significantly moderate the relationship (as suggested by the earlier statistical results), the graph shows that turnover intention is generally higher at all levels of organizational culture when job satisfaction is higher. The highest turnover intention scores are observed in the group with the highest job satisfaction level (6.3865), while the lowest turnover intention scores are seen in the group with the lowest job satisfaction level (4.769). Although the interaction was not statistically significant, this graph may still highlight interesting dynamics for practical insights. It may suggest that high job satisfaction is not necessarily protective against turnover intention in this context, possibly due to other underlying factors that warrant further investigation. For police departments, understanding these nuanced relationships could help refine interventions to retain officers.

DISCUSSION

The findings from this study offer insights into the complex interplay between organizational culture, job satisfaction, and turnover intention among police officers in Malaysia. The results indicate a positive relationship between organizational culture and turnover intention, moderated by varying levels of job satisfaction. Although the interaction effect was statistically non-significant, the graphical analysis revealed that turnover intention appears higher at all levels of organizational culture when job satisfaction is high. This suggests that factors typically expected to reduce turnover intention, such as job satisfaction, may function differently in specific organizational settings, particularly among police officers, due to unique occupational stresses and cultural dynamics (McCreary et al., 2020).

The observed positive association between organizational culture and turnover intention may initially appear counterintuitive, as strong organizational cultures are often linked with lower turnover in many professional settings (O'Reilly, Caldwell, Chatman, & Doerr, 2019). However, in the context of law enforcement, organizational culture may encompass hierarchical structures, rigid regulations, and particular values that, while essential for operational efficiency, can lead to job dissatisfaction or feelings of being undervalued. Police officers often work under stressful conditions with limited autonomy, and such a culture may exacerbate burnout, eventually increasing the desire to leave (Johnson et al., 2021). This finding is consistent with other studies that highlight the unique stressors in policing environments, where the organizational culture may sometimes intensify occupational stress rather than mitigate it (Garcia et al., 2021).

Contrary to expectations, job satisfaction did not significantly moderate the relationship between organizational culture and turnover intention. In typical organizational settings, high job satisfaction serves as a buffer, reducing turnover intentions by enhancing employees' sense of fulfillment and attachment to the organization (Chang et al., 2019). However, the results

Commented [BB7]: Comment. The discussion is brief and does not adequately compare findings with prior literature or explain unexpected results.
Suggestions.
1. Compare your findings with previous studies (e.g., Carlan, 2020; Blau, 2021).
2. Discuss possible explanations for the non-significant moderation effect.
3. Link back to theory and suggest practical implications for police management.
4. Highlight how this study advances knowledge in the field

here suggest that even when police officers report high job satisfaction, they may still experience high turnover intention due to factors inherent to the policing profession, such as the high-stress nature of the job and potential dissonance between personal values and organizational norms. This aligns with findings by Carlan (2020), who reported that satisfaction with certain job aspects, like camaraderie or benefits, might not fully offset the negative impacts of an oppressive or demanding culture.

The non-significant moderation effect suggests that job satisfaction alone may not sufficiently protect against turnover intention in a high-stress occupation like law enforcement. This result supports findings from other studies indicating that, in such professions, additional factors, such as perceived support, mental health resources, and work-life balance, might play more substantial roles in retaining employees (Lambert et al., 2019). The high turnover intention among officers with both high job satisfaction and exposure to certain organizational culture traits implies that job satisfaction in this context may be multifaceted. For example, police officers might experience satisfaction from specific aspects like salary or relationships with colleagues but still hold intentions to leave if they perceive organizational policies or leadership as unsupportive (Van Gelderen et al., 2020).

IMPLICATIONS OF THE STUDY ON UNIVERSITY SUSTAINABILITY

These findings have practical implications for police departments aiming to improve retention. While enhancing job satisfaction through better pay, career advancement opportunities, and team cohesion remains important, it may not be sufficient if the underlying organizational culture contributes to stress or burnout. Leadership could focus on cultivating a supportive culture that values officers' well-being, rather than one solely centered on hierarchical control and strict protocol adherence. For instance, implementing mental health support programs, flexible scheduling, and open communication channels may help reduce turnover intention by addressing officers' broader needs (Shane, 2021). Furthermore, fostering a culture that

emphasizes appreciation, recognition, and shared values could bridge the gap between organizational culture and job satisfaction, potentially mitigating turnover intentions (Trinkner, Tyler, & Goff, 2019).

Police departments could also consider more comprehensive engagement initiatives, such as regular feedback mechanisms that allow officers to voice concerns about organizational culture and suggest improvements. By involving officers in shaping the culture, departments may be able to create an environment that aligns more closely with their values and expectations, thereby reducing turnover intentions (Blau, 2021). This approach would help address specific areas of dissatisfaction, demonstrating the organization's commitment to adapting its culture to meet the evolving needs of its personnel.

LIMITATIONS AND FUTURE DIRECTION

While this study contributes to the literature on organizational culture, job satisfaction, and turnover intention among police officers, there are several limitations and areas for future exploration. Firstly, the cross-sectional design limits the ability to infer causality; longitudinal studies could provide more robust evidence on how organizational culture and job satisfaction influence turnover intention over time. Future research might also benefit from a qualitative approach, such as interviews or focus groups, to gain deeper insights into the specific cultural elements that police officers find challenging. Such methods could uncover nuances that quantitative surveys may overlook, particularly concerning how organizational culture is perceived and experienced at different ranks within the police force.

RECOMMENDATIONS AND CONCLUSION

Another area for future research could involve examining additional moderating variables, such as organizational support, coping mechanisms, or personal resilience, which might influence the relationship between organizational culture and turnover intention (Anshel et al., 2020). For example, researchers could investigate how access to resources like mental health services

or professional development programs impacts officers' desire to stay or leave. Additionally, examining demographic variables, such as age, gender, and years of service, may offer insights into how turnover intention varies across different subgroups within the police force (He et al., 2018).

Given the nonsignificant interaction effect of job satisfaction as a moderator in this study, future research could explore whether other constructs, like organizational commitment or psychological resilience, play more significant roles in this context. Police officers often face intense job demands, and organizational commitment may more directly capture their loyalty or attachment to the department beyond mere satisfaction with specific job aspects. Additionally, resilience as a coping mechanism may buffer against adverse effects of a rigid organizational culture, reducing turnover intention even in high-stress settings (Papazoglou & Andersen, 2019).

CONCLUSION

Commented [BB8]: Comment. The conclusion is too short and lacks actionable recommendations.
Suggestions.
1. Summarize the main findings clearly.
2. Emphasize the practical implications for police departments.
3. Suggest directions for future research (e.g., qualitative exploration of officer experiences).

This study underscores the need to carefully consider the unique occupational context when examining turnover intention among police officers. While job satisfaction is important, it may not be a sufficient buffer against turnover in an environment where organizational culture imposes unique pressures. These findings suggest that police departments should focus on creating a supportive, adaptive culture that prioritizes officers' well-being, potentially enhancing retention. Future research should continue to explore the complex interactions between organizational culture, job satisfaction, and other factors affecting police officers' turnover intentions, providing further guidance for improving retention strategies in this critical profession.

Reference

Abo-Murad, M., Abdullah, A. L. K. Turnover culture and crisis management: Insights from Malaysian hotel industry. Academy of Strategic Management. 2019.

Al-Shurafat, M. S., & Halim, B. B. A. A review of organizational culture and organizational commitment. IOSR Journal of Business. 2018.

Anshel, M. H., Umscheid, D., & Brinthaupt, T. M. Modifying occupational stress: A review of police officers' challenges and mental health strategies. Psychology & Health. 2020; 35(8), 1014-1036.

Blau, G. Exploring the impact of emotional labor on police burnout and turnover intention. Criminal Justice Studies. 2021; 34(2), 189-205.

Carlan, P. E. Job satisfaction among police officers: The role of organizational culture and work environment. Journal of Criminal Justice. 2020; 44, 85-92.

Chong, K. Y., & Mui, H. K. The importance of integrity practices on task performance Royal Malaysia Police: The mediating role of organizational commitment and job satisfaction. International Journal of Social Science Research. 2021.

Francis, B. R., Ahmad, R., & Abdullah, S. M. The dimension of performance appraisal justice and intention to quit work: The mediating role of organizational support. ResearchGate. 2021.

Garcia, A., Wang, H., & Cohen, B. Understanding the role of organizational culture in police stress and turnover intention. Journal of Policing and Criminal Psychology. 2021; 36(1), 56-68.

Ghazali, H., & Asmawi, N. Work culture and turnover intention: A case of restaurant employees in Putrajaya, Malaysia. Journal of Academic Research in Business. 2020.

Hassan, N., Wahab, I. A., Halif, M. M., & Ali, S. Leadership styles and employee satisfaction among police officers. Springer. 2018.

He, N., Zhao, J., & Archbold, C. Gender and police stress: The impact of gendered socialization and occupational stressors. International Journal of Police Science & Management. 2018; 20(3), 243-251.

Ismail, A., Ghani, A. B. A., & Subhan, M. Job satisfaction and turnover intentions of police officers in Malaysia. Semantic Scholar. 2023.

Jabeen, N., Omar, M. K., & Aluwi, A. H. Ethical leadership and organizational culture in police retention. ResearchGate. 2019.

Johnson, L., Meng, Y., & Parker, S. Police stress and job satisfaction: A review of the moderating role of organizational culture. Journal of Applied Psychology. 2021; 106(6), 936-952.

Kamaluddin, M. R., Mat Basir, S., & Bujang, M. A. Impacts of workplace culture on deviant workplace behavior: A systematic review. SAGE. 2024.

Lambert, E. G., Hogan, N. L., & Barton, S. M. Work environment and turnover intention among correctional staff. Criminal Justice and Behavior. 2019; 46(8), 1138-1153.

McCreary, D. R., Thompson, M. M., & Pastorek, M. Law enforcement organizational stress. Policing: An International Journal. 2020; 43(4), 762-778.

Narandaran, H. R. Stress and job satisfaction among school teachers in Malaysia. ProQuest. 2018.

Narandaran, H. R. Stress and job satisfaction among school teachers in Malaysia: The association with the psychosocial working environment and organizational justice. ProQuest. 2018.

Omar, M. K., Aluwi, A. H., & Hussein, N. Factors influencing job stress among Malaysian traffic police officers. IOSR Journal of Financial Research. 2020.

O'Reilly, C. A., Caldwell, D. F., Chatman, J. A., & Doerr, B. Organizational culture and retention: Insights for police departments. Administrative Science Quarterly. 2019; 64(1), 2-20.

Papazoglou, K., & Andersen, J. P. Police officer resilience and stress management. Policing: A Journal of Policy and Practice. 2019; 13(4), 475-488.

Saad, S., & Rashid, N. M. Police officers' intentions for early retirement in Kedah contingent. UUM eThesis. 2023.

Shane, J. M. Improving law enforcement management: Effective strategies to mitigate stress and enhance job satisfaction. Police Quarterly. 2021; 24(3), 312-328.

Sulaiman, S. B. The role of ethical leadership, monetary reward, and career opportunity on intention to stay among auxiliary police. UUM eThesis. 2024.

Trinkner, R., Tyler, T. R., & Goff, P. A. Organizational justice and support in law enforcement. Law and Human Behavior. 2019; 43(2), 99-110.

Van Gelderen, B., van der Heijden, B., & Witte, H. D. Predictors of burnout and turnover intention among police personnel. Journal of Occupational Health Psychology. 2020; 25(4), 210-221.
