# Supplementary material for: Job satisfaction as a moderator between organizational culture and turnover intention among Malaysian police officers
Source: PLoS One. 2025 Sep 2;20(9):e0331458. doi: 10.1371/journal.pone.0331458 (PMC12404360; doi:10.1371/journal.pone.0331458)
Supplement: Attachment — Submitted filename: Response to reviewer.docx [file pone.0331458.s002.docx]

# Response to Reviewers and Detailed Change Log

Manuscript ID: PONE-D-25-23324

Title: Job satisfaction as a moderator between organizational culture and turnover intention among Malaysian police officers

**Dear Editors and Reviewers,**

We thank the Editors and both reviewers for their thorough and thoughtful feedback on our manuscript. We have carefully addressed all points raised and made substantial revisions to improve the clarity, rigor, and presentation of our work. Below we provide a detailed, point-by-point response to the comments from Reviewer #1 and Reviewer #2, followed by a detailed change log indicating where the corresponding revisions were made in the manuscript.

| **Reviewer** | **Comment** | **Response** |
| --- | --- | --- |
| **1** | **1 :** The role of job satisfaction as a moderator is not clearly presented throughout the manuscript. | Thank you for the insightful comment. We have thoroughly revised the manuscript to ensure that the role of job satisfaction as a moderator is clearly and consistently presented across all major sections, including the Abstract, Introduction, Theoretical Framework, Research Hypotheses, Methodology, Results, Discussion, and Conclusion. Specific updates include theoretical justification (Lines 99–102), detailed moderation hypothesis (Lines 180–190), and clear results reporting (Lines 270–295). Please refer to Track Changes in the revised manuscript. |
|  | **2.** The manuscript lacks a clearly defined hypothesis section. | We appreciate this observation. In response, we have added a dedicated section titled '5. Research Hypotheses' (Lines 190–210) that explicitly states three key hypotheses derived from the theoretical framework. These hypotheses clarify the direct and moderating effects examined in the study. |
|  | **3.** Simple slope analysis and visual graphs were presented despite non-significant moderation effects. | Response: Thank you for your observation. We acknowledge that the moderation effect was not statistically significant (p = .117). However, we retained the simple slope analysis and visual interaction plot (Figure 2) to provide a clearer interpretation of the trend, as this is common in moderation analysis practice. Although not significant, the visualization assists readers in understanding the conditional nature of the interaction and supports transparency in statistical reporting. This decision is aligned with previous empirical practice and enhances clarity in communicating complex results. |
|  | 4. Remove discussion on leadership and ethics (Lines 133–137) as they are outside the study   scope. | We appreciate the reviewer’s comment and have removed the unrelated discussion on leadership and ethics in the revised manuscript. Specifically, lines 133–137 in the previous version have been omitted to maintain a focused discussion aligned with the study’s objectives and scope. |
|  | 5. Consider the profile of the respondents when interpreting the non-significant moderation   effect. | Thank you for this insightful suggestion. We have updated the discussion section to consider how respondents’ profiles—such as rank, tenure, and departmental affiliation—might explain the non-significant moderation effect. These demographic factors may influence perceptions of organizational culture and satisfaction, thus impacting the moderation mechanism. The revised discussion (Paragraph 45–47) reflects these considerations to strengthen contextual interpretation. |
| **2** | 1. The Abstract suggests counterintuitive findings and requires clearer language. | We appreciate this feedback. The abstract has been fully revised to follow a clearer and more conventional structure—comprising background, objectives, methods, key findings, and implications. We clarified that while both organizational culture and job satisfaction were significantly related to turnover intention, the hypothesized moderation effect was not supported. Technical terms have been reduced for accessibility (Lines 29–49). |
|  | 2. The manuscript lacks a strong theoretical foundation. | Thank you for your valuable input. We strengthened the theoretical foundation by elaborating on Social Exchange Theory and the Job Demands-Resources (JD-R) Model. The revised theoretical framework (Paragraphs 15–17; Lines 164–180) clearly explains the rationale behind the hypotheses and how these theories underpin the conceptual relationships between variables. Citations from Francis et al. (2020), Johnson et al. (2021), and other recent empirical studies were added to bolster theoretical grounding. |
|  | 3. Clarify the sampling technique and provide demographic distribution. | We have revised the Methodology section (Paragraphs 18–21; Lines 184–215) to explicitly explain the stratified sampling method used and how participants were selected across ranks and departments. Table 1 now presents the full demographic profile of respondents, including gender, age, rank, and department. |
|  | 4. The directionality of the findings is counterintuitive and needs better interpretation. | Thank you for highlighting this. We have addressed the counterintuitive positive associations in the discussion section (Paragraphs 42–45). We now offer theoretical and contextual explanations, including cultural rigidity, perceived hierarchy, and misalignment with individual values, to interpret the findings more thoroughly. |
|  | 5. More explanation is needed on how high job satisfaction may coexist with high turnover intention. | We agree with the reviewer. The revised discussion explores how satisfied officers may still express turnover intention due to career stagnation, unmet expectations, or institutional misalignment. We draw on findings from McElroy et al. (1999) and Carlan (2007) to substantiate this paradox (Paragraph 44). |
|  | 6. Ensure figures and tables follow formatting guidelines. | We have ensured that all figures and tables now comply with PLOS ONE formatting guidelines. Tables are referenced in the text, and figure captions are descriptive and appropriately labeled. Table 1 and Figure 1 have been adjusted accordingly. |

1. Tracked Changes Manuscript

We have uploaded a revised version of the manuscript with all changes clearly highlighted using Microsoft Word Track Changes, as well as a clean version for your review.

2. Data Availability Statement

A complete data availability statement has been added both in the manuscript and within the online submission form, stating:
"All data are in the manuscript and/or supporting information files."

3. In-text References to Tables

The manuscript has been updated to include explicit in-text references to Table 1 and Table 2, in accordance with production guidelines.

4.	Reference List Formatting

The reference list has been revised and renumbered to follow the PLOS ONE Vancouver citation style, ordered by appearance.

5.	Ethics Statement Placement

The ethics statement now appears only in the Methods section, as requested. All redundant instances have been removed.

6.	Ethics Approval Documentation

We have uploaded the original ethical approval letter issued by the Universiti Kebangsaan Malaysia Research Ethics Committee (Reference number: JEP-2024-230), along with its English translation.

We hope these revisions are satisfactory and we respectfully submit our revised manuscript for your further consideration.

Thank you for your time and support.

Sincerely,
Erny Yusnida binti Che Yusoff

Faculty of Social Sciences and Humanities

The National University of Malaysia

[orcid Detail '0000-0002-3065-4078']
